# Weaning management in a patient with heart dysfunction: a case report

**DOI:** 10.3389/fphys.2025.1609975

**Published:** 2025-06-02

**Authors:** Haobo Jiang, Meiling Lao, Weixian Xu, Yunhai Zhang

**Affiliations:** Department of Critical Care Medicine of Foshan Hospital of Traditional Chinese Medicine, Foshan, China

**Keywords:** weaning, spontaneous breathing trial, T-piece, pressure support, weaning-induced pulmonary edema

## Abstract

**Background:**

Patients undergoing weaning from mechanical ventilation face the risks of reintubation. Spontaneous breathing trials (SBTs), including T-piece (SBT-T) and pressure support (SBT-P), are commonly used to assess extubation readiness. Current guidelines favor the use of SBT-P. Weaning-induced pulmonary edema (WIPO) is common after extubation, which could lead to extubation failure.

**Case:**

We report the case of a patient on ventilation who failed the first extubation attempt following successful SBT-P due to WIPO. SBT-T was implemented for the patient in the second weaning attempt.

**Methods:**

During the subsequent SBT-T, signs of WIPO recurred. Instead of terminating the trial, we managed the patient with intensive monitoring, fluid management, and blood pressure control.

**Result:**

After targeted interventions, this patient was successfully extubated during the second weaning attempt.

**Conclusion:**

This case highlights the utility of SBT-T in unmasking WIPO risk in weaning patients with cardiac dysfunction. By enabling proactive management during the trial, SBT-T may enhance safety in high-risk populations.

## Introduction

Early weaning from mechanical ventilation is critical to reduce complications such as ventilator-associated pneumonia. During this process, two major methods of spontaneous breathing trial (SBT) would be considered to assess the patient’s readiness to be weaned from the ventilator: T-piece (SBT-T) and pressure support (SBT-P). Current guidelines recommend use of SBT-P over SBT-T for assessing extubation readiness. However, there is still the possibility for extubation failure. Here, we present a case to illustrate how we chose the SBT methods and share our opinions on the weaning process.

## Case presentation

This case report is approved by the Clinical Medical Research Ethics Committee of Foshan Hospital of Traditional Chinese Medicine (KY [2025]080). The writing of the report meets local legislation and institutional requirements. Participants have provided their written informed consent to participate in this study. Written informed consent was obtained from the individual for the publication of any potentially identifiable figures or data included in the article. The figures and data are anonymized.

A 73-year-old man with a history of hypertension was admitted to the orthopedics department on 18 March 2022, following a motor vehicle accident, resulting in a right calf fracture. He underwent emergency open reduction and internal fixation for fracture stabilization. During postoperative care, the patient developed acute myocardial infarction, leading to acute left heart failure (HF) and hypoxemia. The patient’s family declined coronary artery intervention, prompting a transfer to the intensive care unit (ICU) on March 21 for advanced monitoring and supportive management.

Prior to this hospitalization, the patient had been on regular antihypertensive therapy with no documented pulmonary dysfunction. He had a negative family history for cardiovascular diseases. Baseline echocardiography at admission revealed preserved systolic and diastolic cardiac function.

## Physical examination

The patient exhibited shortness of breath. Wheezing sound and coarse rales were found in bilateral lungs. There was no apparent audible murmur in cardiac auscultation.

## Imaging and diagnostic findings

The following values were observed: troponin I: 12.892 ug/L; N-terminal pro-B-type natriuretic peptide (NT-proBNP): 9853.0 pg/mL. Analysis of blood and gas (ABG) yielded the following: pH 7.439, PaCO_2_ 33.1 mmHg, PaO_2_ 67.7 mmHg. Electrocardiography suggested acute anterior myocardial infarction. Chest X-ray suggested bilateral pulmonary edema.

## Treatment

The patient underwent endotracheal intubation for mechanical ventilation after failure of non-invasive measures on the day of admission. Subsequently, he developed hospital-acquired pneumonia. Following 25 days of ventilator support and targeted treatment, his clinical condition improved: infection was controlled, hemodynamics stabilized, and oxygenation (PaO_2_/FiO_2_) exceeded 200 mmHg, prompting consideration for weaning.

On April 15, after sedation withdrawal, the patient regained consciousness and effective cough reflex, passing the cuff-leak test. SBT-P was initiated with 6 cmH_2_O pressure support (PS) and 5 cmH_2_O positive end-expiratory pressure (PEEP). During the 2-h SBT-P, he remained hemodynamically stable. The following were observed: PaO_2_: 123 mmHg; PaCO_2_: 42 mmHg; oxygenation index (OI, PaO_2_/FiO_2_): 308; heart rate (HR): 80–90/min; respiratory rate (RR): 18/min; and blood pressure (BP): 110–120/80–90 mmHg. Extubation was implemented, and a high-flow nasal cannula (HFNC) was initiated at 50% FiO_2_ and 50 L/min flow rate.

Within 2 h, the patient developed respiratory distress (HR: 118/min; BP: 153/78 mmHg; RR: 30/min; SpO_2_: 93%). Despite escalating HFNC parameters, hypoxia persisted, accompanied with bilateral wheezing and coarse crackles. Non-invasive ventilation (NIV) was attempted but failed due to agitation and poor cooperation. Concerns over impaired secretion clearance prompted reintubation to ensure respiratory stability.

**FIGURE 1:**
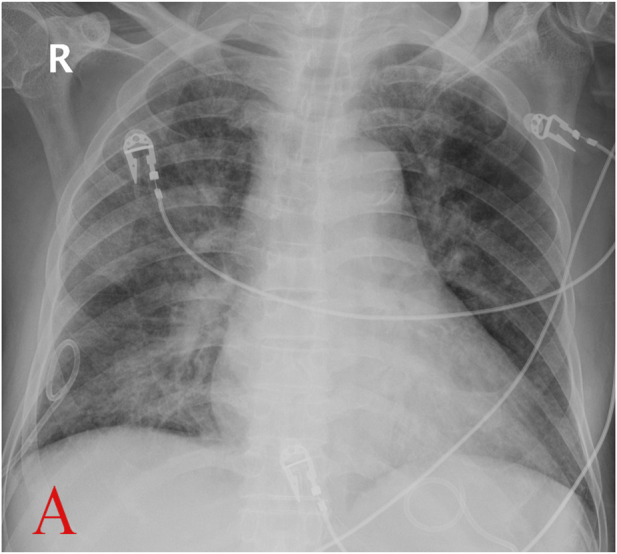
Chest X-ray before extubation (A, Apr.14) (with permission from the patient.)

**FIGURE 2:**
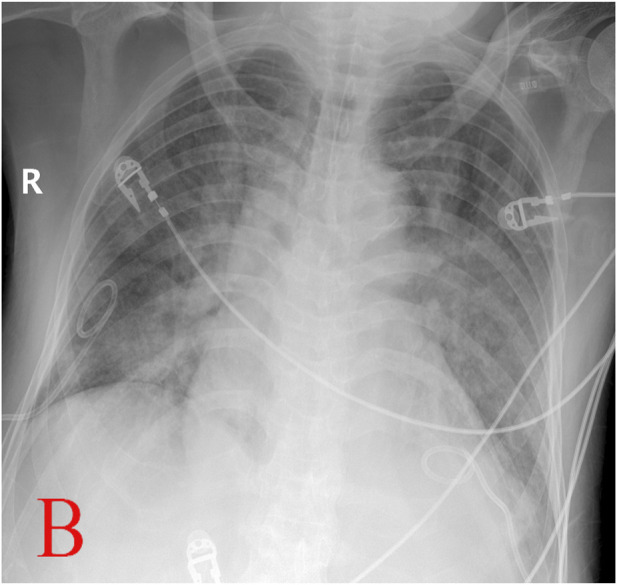
Chest X-ray after reintubation (B, Apr.15) (with permission from the patient.)

**FIGURE 3:**
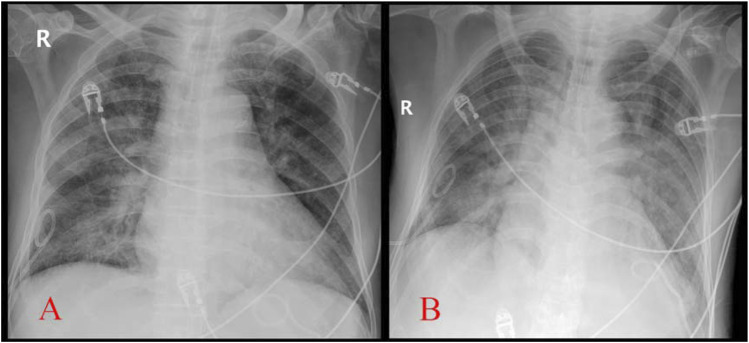
Comparison of chest X-ray before extubation (A, Apr.14) and after reintubation (B, Apr.15) (with permission from the patient.)

Fiber-optic bronchoscopy revealed copious amounts of pink frothy sputum in both lungs, suggesting pulmonary edema. Invasive mechanical ventilation restored stability after sputum clearance. NT-proBNP levels increased significantly post-extubation: 6,572 pg/mL vs. 1,653 pg/mL (before extubation). Serial chest X-rays demonstrated progressive bilateral patchy opacities (Figures 1–3).

We concluded that weaning-induced pulmonary edema (WIPO) occurred after extubation. By April 17, respiratory function, oxygenation (OI: 428), and hemodynamics stabilized under ventilator settings (PS: 6 cmH_2_O; PEEP: 5 cmH_2_O; same assist level as SBT before the previous extubation). Vital signs included HR: 95/min, BP: 103/58 mmHg, and RR: 17/min. ABG showed PaO_2_: 171 mmHg and PaCO_2_: 52 mmHg. We elaborated the following process to the patient. After reaffirming cuff-leak test eligibility and obtaining informed cooperation, SBT-T commenced at 10:00 a.m.

Initially, the patient was calm and tolerated SBT-T well. At 2 h, SpO_2_ declined to 97% (from 100%) and failed to recover over 30 min. We presumed early WIPO impairing gas exchange in alveoli, resulting in the decrease in SpO_2_. We intervened immediately instead of continuous monitoring. Intravenous furosemide (20 mg) was administrated to the patient to achieve rapid negative fluid balance.

**FIGURE 4:**
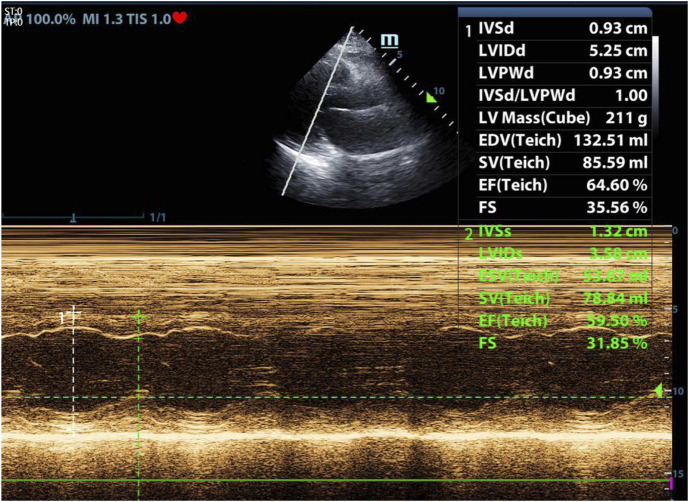
Echocardiography during SBT-T after reintubation. Left ventricular ejection fraction (LVEF): 59.5% (with permission from the patient.)

**FIGURE 5:**
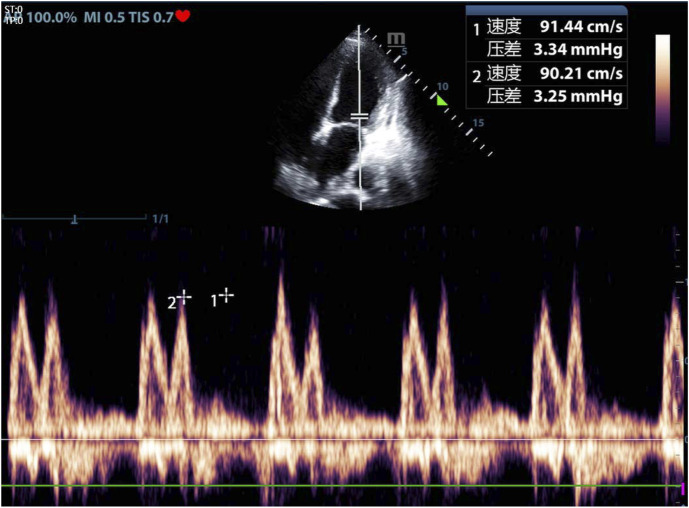
Echocardiography during SBT-T after reintubation. E/A: 1.0, E/e’: 16.2 (with permission from the patient.)

By 15:00, the fluid volume reached approximately 800 mL negative after diuresis. The patient was a little agitated but still cooperative. Vital signs included RR: 18/min, HR: 109/min, and BP: 143/74 mmHg. ABG showed PaO_2_: 92 mmHg, OI: 249, and PaCO_2_: 43 mmHg. We checked echocardiography for the patient (Figures 4, 5).

Dexmedetomidine infusion was administrated to mitigate agitation, targeting the Richmond Agitation–Sedation Scale: -2 to 0. His systolic blood pressure (SBP) was strictly controlled below 130 mmHg by infusion of sodium nitroprusside.

Over the subsequent 3 h, the patient remained stable. The vital signs at 6:00 p.m. were as follows: HR 50–70/min, SBP 120–130 mmHg, RR 13–16/min, SpO_2_ 100%. ABG showed PaO_2_: 111 mmHg, OI: 300, and lactate: 0.8 mmol/L. Cumulative fluid balance reached 1,000 mL negative from 8:00 a.m. Because the oxygenation became stable, we believed diuresis was no longer necessary. The patient was awake, and his cough reflex was effective; then, he was extubated. Post-extubation NT-proBNP was 3,443 pg/mL vs. 2,988 pg/mL (before the SBT-T). Negative fluid balance was maintained for days because of elevated NT-proBNP, stable hemodynamics, and normal lactate. The patient was transferred from the ICU without respiratory support later, marking a successful outcome.

### Patient perspective

The patient later reported, “I really felt very uncomfortable after the first extubation. Horrible, and severely short of breath. But much better in the second time. They comforted me and told me what I should do. Breathing as normal and trying to keep peaceful. I just followed their instructions. Much more comfortable. No overt dyspnea.”

## Follow-up and outcome

The patient recovered well after medical treatment in the cardiovascular department. He receives follow-up treatment in clinic until now.

## Discussion

Prolonged mechanical ventilation (27 days in this case) carries significant risks, including ventilator-associated pneumonia, prolonged hospitalization, increased medical cost, and mortality (Cheng et al., 2011). This patient luckily survived. Early extubation is critical, but approximately 24.5% of patients experience extubation failure (Schmidt et al., 2016), leading to increased morbidity and mortality (Frutos-Vivar et al., 2011; Gao et al., 2016). SBT is recommended to assess extubation readiness. So far, low-level pressure support (5–8 cmH_2_O) and T-piece are the major methods for SBT (Thille et al., 2020a). The current guideline by the American College of Chest Physicians/American Thoracic Society prefers SBT-P to SBT-T (Ouellette et al., 2016) based on three clinical trials, which concluded that extubation success by SBT-P was higher and that SBT-P was associated with a shorter duration of ventilation (Pellegrini et al., 2016; Subirà et al., 2019). However, we do not totally agree with this recommendation. Recently, a meta-analysis study demonstrated no superiority of SBT-P over SBT-T in predicting successful weaning (Li et al., 2020). In our opinion, SBT-P might not be suitable for all kinds of patients in different clinical settings. SBT-P reduces respiratory muscle workload by offsetting the resistance of the airway and endotracheal tube, thus decreasing oxygen consumption of the respiratory muscle (Sklar et al., 2017), but it potentially overestimates respiratory function or covers some underlying risks, e.g., WIPO.

In patients with chronic obstructive pulmonary disease (COPD), heart disease, or obesity, weaning failure for WIPO could reach 60% (Liu et al., 2016). Our patient is one of them. When a patient is disconnected from the ventilator (both SBT-T and extubation), sudden transition from positive pressure ventilation to spontaneous breathing with negative inspiratory intra-thoracic pressure leads to abruptly increased venous return. If such rapidly increased cardiac loading exceeds the compensatory capability of the heart, namely, overload, WIPO would occur. In addition, weaning from the ventilator causes an extra burden for both respiratory and cardiovascular systems, which would augment oxygen demand for the myocardium and respiratory muscle. Myocardial ischemia, decreased LV compliance, positive fluid balance, and acute hypertension induced by weaning frequently take part in the process (Perren and Brochard, 2013; Teboul, 2014). All of these mechanisms of cardiac origin could result in the eventual weaning failure, which is presented as WIPO. In clinical practice, low-level (approximately 5 cmH_2_O) PEEP is always used to prevent WIPO in SBT-P. However, this measure might mask the risk because the patient has to confront a setting without PEEP after extubation. Our patient did not show any signs of WIPO in SBT-P but failed the first extubation attempt as a result of WIPO.

We chose SBT-T for the patient in the second weaning attempt because SBT-T could replicate post-extubation physiology, such as the work of breathing, when the patient is totally disconnected from the machine (Sklar et al., 2017). SBT-T seems to more sensitively evaluate the patients’ spontaneous breathing tolerance without machine support (Frutos-Vivar and Esteban, 2014; Kuhn et al., 2016).

A systematic search of PubMed, Embase, and Cochrane databases was conducted using the terms “spontaneous breathing trial,” “pressure support,” and “T-piece.” From 24 eligible English-language articles, nine clinical trials comparing SBT-P (pressure support) and SBT-T (T-piece) techniques were selected for analysis (Table 1). Three trials demonstrated superior extubation success rates with SBT-P, while the remaining six showed no statistically significant differences between the methods.

**TABLE 1:** Clinic trials for SBT-P vs. SBT-T.

Author	Year	Amount (P/T)	More successful extubation	Less ventilator-free days
Thille, A.W. (Thille et al., 2022)	2022	484/485	n	n
Subirà, C. (Subirà et al., 2019)	2019	575/578	P	-
Na, S.J. (Na et al., 2022)	2022	314/473	n	-
Thille, A.W. (Thille et al., 2020b)	2020	243/398	P	-
Zhang, B. (Zhang and Qin, 2014)	2014	93/115	n	-
Chittawatanarat, K. (Chittawatanarat et al., 2018)	2018	260/260	n	-
Brochard, L. (Brochard et al., 1994)	1994	31/35	P	-
Teixeira, S.N. (Teixeira et al., 2015)	2015	46/66	n	n
Vitacca, M. (Vitacca et al., 2001)	2001	26/26	n	n

Abbreviations: P, spontaneous breathing trial by pressure support; T, spontaneous breathing trial by T-piece; n, no significance; -, no mention.

Notably, existing comparative studies primarily evaluate these SBT modalities as predictors of extubation outcomes rather than their efficacy in facilitating the weaning process itself. Our critical appraisal revealed a consistent limitation across these trials: inadequate stratification of participants by specific disease etiologies. This oversight acquires clinical relevance in this case, where the patient experienced initial weaning failure. We attribute this failure to WIPO, which is supported by fiber-optic bronchoscopy findings, radiographic evidence, and a marked post-extubation elevation in NT-proBNP. The pathophysiological basis for this interpretation lies in the cardiac stress response: myocardial stretch from excessive preload (e.g., volume overload and increased venous return) and/or afterload (e.g., hypertension) triggers BNP release to promote vasodilation and diuresis (Boomsma and Meiracker, 2001). As the gold-standard biomarker for LV dysfunction, BNP elevation during SBT strongly suggests decompensated HF secondary to WIPO (Mekontso et al., 2014). While point-of-care BNP testing was unavailable in our unit, the observed NT-proBNP surge provided compelling surrogate evidence of cardiac dysfunction to corroborate our clinical conclusion.

Given the physiological rigor of SBT-T, we propose SBT-T as the preferential modality for high-risk patients susceptible to WIPO. Due to the limited availability of rapid NT-proBNP testing in our clinical setting, bedside ultrasound was implemented during the second weaning attempt to monitor hemodynamics following SBT-T initiation. Slight deterioration of vital signs during the trial prompted echocardiographic evaluation, which revealed preserved LVEF (59.5%) but diastolic impairment (E/e’ ratio: 16.2). This hemodynamic profile aligns with the pathophysiological pattern described by Sanfilippo et al. (2020), wherein elevated E/e’, a validated surrogate for LV filling pressure, serves as a critical indicator of diastolic dysfunction. These findings implicate that WIPO for this patient was associated with impaired LV diastolic function, instead of systolic dysfunction.

The management of WIPO parallels principles for acute HF, prioritizing diuresis to reduce cardiac preload secondary to increased venous return. While Song et al. (2024) advocated prophylactic diuretic administration prior to SBTs, our clinical observations challenge this approach. Under positive intrathoracic pressure, hemodynamic equilibrium may mask fluid overload until ventilator disconnection triggers WIPO manifestation. Premature systematic fluid removal without clear therapeutic targets risks iatrogenic complications (e.g., hypokalemia and hypoperfusion). Consequently, our protocol reserves diuretics for confirmed WIPO during SBT-T, discontinuing therapy upon resolution of clinical indicators (e.g., SpO_2_ stabilization). Post-episode management maintains a daily negative fluid balance (guided by serial NT-proBNP trends), unless lactate elevation or hemodynamic instability contraindicates this strategy, until NT-proBNP starts to decrease.

Afterload reduction becomes imperative if hypertension accompanies WIPO. Sodium nitroprusside supersedes alternative vasodilators (e.g., urapidil and nitroglycerin) in our practice due to its rapid, titratable antihypertensive efficacy.

Sympathetic hyperactivity during weaning warrants concurrent mitigation. Elevated urinary vanillylmandelic acids (the end product of catecholamine metabolism) during SBT corroborate the elevated plasma catecholamine levels in these patients (Koksal et al., 2003). Aligning with Philippe’s (Vignon, 2023) recommendation for beta-blocker, a negative inotropic and chronotropic drug, in the setting that adrenergic response to SBT induces hypertension, we employed a new sedative, dexmedetomidine (a highly selective α_2_ adrenergic receptor agonist in the central nervous system), during our patient’s second weaning attempt. Its dual anxiolytic and sympatholytic properties optimally addressed concurrent anxiety, sinus tachycardia, and hypertension. Our opinion against routine inotropes in WIPO without systolic dysfunction aligns with consensus evidence provided by Sanfilippo et al. (2020) and Caille et al. (2010), even with any previous history of HF.

According to established protocols, SBT termination is mandatory upon detection of WIPO, an indicator of weaning failure (Mezidi et al., 2022). However, we maintained the SBT-T, which simulated the post-extubation physiological conditions for management. Conventionally, SBT durations range from 30 to 120 min, although the appropriate one has not yet been defined (Na et al., 2022). We extended this period to 8 h in our patient. This prolonged duration raises valid concerns regarding potential respiratory muscle fatigue and endotracheal tube discomfort, but it was necessary for observation and intervention. Three additional factors justified our approach: first, the absence of chronic lung diseases (e.g., COPD) that causes predisposition to respiratory muscle weakness eliminated a key risk factor for fatigue. Second, the use of an 8-mm inner diameter endotracheal tube minimized airway resistance. Third, comprehensive patient education fostered cooperation throughout the extended trial. Successful weaning ultimately validated this tailored strategy.

## Conclusion

Our clinical observations indicate that not all HF patients develop WIPO during weaning. This variability likely reflects that WIPO is a complex pathophysiology of cardiopulmonary interactions. However, patients with preexisting HF exhibit high WIPO risk due to impaired LV compliance, which is a hallmark of cardiac dysfunction.

By simulating post-extubation physiology, SBT-T serves early detection of WIPO while maintaining ventilator accessibility in this population. Notably, SBT-T continuation during WIPO manifestation permits observation and intervention. The maintained ventilator interface allows immediate respiratory support easily if necessary, thus significantly reducing reintubation requirements and enhancing clinical safety.

## Data Availability

The original contributions presented in the study are included in the article/Supplementary Material; further inquiries can be directed to the corresponding author.
